# The Tuberculosis-Depression Syndemic and Evolution of Pharmaceutical Therapeutics: From Ancient Times to the Future

**DOI:** 10.3389/fpsyt.2021.617751

**Published:** 2021-06-01

**Authors:** Martie Van Der Walt, Karen H. Keddy

**Affiliations:** Tuberculosis Platform, South African Medical Research Council, Pretoria, South Africa

**Keywords:** history, isoniazid, psychiatry, antidepressant, policy, syndemic, depression, tuberculosis

## Abstract

The interplay between tuberculosis and depression has been problematic since the humoralists. Over the centuries similarities in disease management have transpired. With the advent of isoniazid chemotherapy, transformation of tuberculosis patients from morbidly depressive to euphoric was noted. Isoniazid was thereafter widely prescribed for depression: hepatotoxicity ending its use as an antidepressant in 1961. Isoniazid monotherapy led to the emergence of drug resistant tuberculosis, stimulating new drug development. Vastly increased investment into antidepressants ensued thereafter while investment in new drugs for tuberculosis lagged. In the 21st century, both diseases independently contribute significantly to global disease burdens: renewed convergence and the resultant syndemic is detrimental to both patient groups. Ending the global tuberculosis epidemic and decreasing the burden of depression and will require multidisciplinary, patient-centered approaches that consider this combined co-morbidity. The emerging era of big data for health, digital interventions and novel and repurposed compounds promise new ways to treat both diseases and manage the syndemic, but absence of clinical structures to support these innovations may derail the treatment programs for both. New policies are urgently required optimizing use of the current advances in healthcare available in the digital era, to ensure that patient-centered care takes cognizance of both diseases.

## Introduction

Tuberculosis contributes considerably to human disease burdens. In 2018, the estimated incidence was 10.0 million cases globally, with 1.45 million deaths (1); the estimated prevalence, including latent infection, was 1.93 billion in 2017 (2). Globally, the highest disease burdens were reported from South-East Asia (44%) – 27% of cases occurring in India – and Africa (24%) (1).

Depression is forecast to be the greatest contributor to global disease burdens by 2030 (3). Official World Health Organization estimates for the lifetime prevalence of depressive disorders are 10–15%, affecting over 120 million people world-wide (4). From 1990 to 2007, the prevalence of major depression increased by 33.4%, increasing a further 14.3% over the following 10 years (2), particularly affecting South Asia (predicted point prevalence [PPP] of 8.6), Africa and the Middle East (PPP of 6.6), the lowest incidence reported from North America (PPP of 3.7) (5). Global deaths associated with major depression were estimated in excess of 2.2 million in 2010 (6), with a 20-fold greater suicide risk than the general population (4). Unlike tuberculosis (1), comprehensive management policies for depression are not universally available (7).

Meta-analysis of data from low-and middle-income countries (LMICs) suggests adult tuberculosis patients have 1.98, 1.75, and 3.68 higher odds for subsyndromal depression, brief depressive episodes, and depressive episodes, respectively, compared to adults without tuberculosis (8). Nonetheless, past wisdom on disease associations was lost in the latter half of the 20th century, as chemotherapeutics developed in parallel, allowing patient-centered management to diverge. This review highlights the syndemic, presenting potential policy solutions offered by the digital era, arguing that clinical structures should be developed permitting patient-centered co-management of tuberculosis and depression.

## The Interaction Between Tuberculosis and Depression

Singer et al. have defined the criteria for a syndemic:

(1) “Two (or more) diseases or health conditions cluster within a specific population;
(2) Contextual and social factors create the conditions in which two (or more) diseases or health conditions cluster; and
(3) The clustering of diseases results in adverse disease interaction, either biological or social or behavioral, increasing the health burden of affected populations” (9).

The syndemics of HIV and tuberculosis and HIV and depression are well-recognized (10), but that of tuberculosis and depression lags behind both. Up to 70% of tuberculosis patients may present with depression (11, 12), associated with poor healthcare seeking behavior and disengagement from treatment, leading to poorer outcomes, including loss to follow-up and death (13). Tuberculosis and depression share common risk factors, including homelessness, HIV co-infection, and alcohol and substance dependency (13): depression thus increases the risk for acquiring tuberculosis. Successful treatment of antimicrobial susceptible tuberculosis requires patients take daily treatment consistently for 6 months to prevent acquisition of drug resistance: co-administration of antituberculotics with antidepressants may contribute to patients' discontinuing treatment due to side effects of co-therapy (11). Some antituberculotics can have serious psychiatric side effects, exacerbating the depressive state (11). Certain antidepressants can decrease the bioavailability of antituberculotics, or vice-versa, due to common metabolic pathways; rifampicin for instance is a powerful inducer of cytochrome P450 in the liver (11). Conversely, isoniazid (INH) and linezolid, having monoamine oxidase inhibitor (MAOI) activity, may increase serotonin bioavailability, if given with selective serotonin reuptake inhibitors (SSRIs), causing serotonin syndrome (11). INH may potentiate hepatic failure in patients with preceding liver damage (11).

## Past Wisdom, Tuberculosis, and Melancholia

The Ancient Greeks and Roman humoralists described the interplay between tuberculosis and depression: Hippocrates (460-370 BCE) attributed diseases to imbalances between the four bodily fluid components (14), theorizing a predominance of black bile resulted in melancholia and “fatal pulmonary” disease (15). Early Sanskrit writings similarly included mental diseases among the causes of “consumption” (15).

Prior to the 19th century, tuberculosis patients were predominantly managed at home or hospitalized (16). Care of the mentally ill was the responsibility of relatives and friends; those considered dangerous or disruptive were dealt with by the community, being whipped out of town or more rarely admitted to general hospitals (17). The 17th century Cartesian view of melancholia (depression) is noteworthy as Descartes believed it was rooted in the soul as the seat of human passions, although Descartes expounded the close linkages between the soul and the body, while promoting sleep, exercise and specific diets to “restore the balance of the humors” (18).

By the 19th century, both diseases were treated through institutionalization: sanatoria for tuberculosis patients and asylums for patients with severe mental illness (16, 17). Treatment of mental diseases was relatively crude and options limited. In the early 19th century, a London Hospital superintendent wrote: “confinement or restraint may be *imposed as a punishment* with some advantage, and on the whole, [he considered] *fear the most effectual principle by which to reduce the insane to orderly*” (our italics) (17). By the mid-19th century, recognition of the infectious nature of tuberculosis meant management evolved to specialized sanatoria offering bedrest, nutrition, and sunshine (19). “Classes” for tuberculosis patients unable to access sanatoria were introduced in 1905 by Joseph Pratt, a Boston physician, supported by “friendly visitors” – trained nurses functioning as social workers (initiating the hospital social work program) (20). In Pratt's assessment, he “never fully realized at the time the great influence of [their] class meetings in giving new patients courage and hope. Many of them never could have been induced to follow the strict rest treatment if they had not seen with their own eyes others who had regained their health by doing it” (20).

Pratt extended the concept to enhance psychotherapy programs for patients with anxiety neuroses in the early 1930s, founding, with others introducing psychiatric group therapy (20). Whether due to the emerging era of 20th century super-specialization or to narrowing of management foci, references to the association between tuberculosis and mental illness waned after the mid-1950s; only in recent years has the syndemic regained recognition (21).

## The Development of Antituberculotics

**Figure 1:**
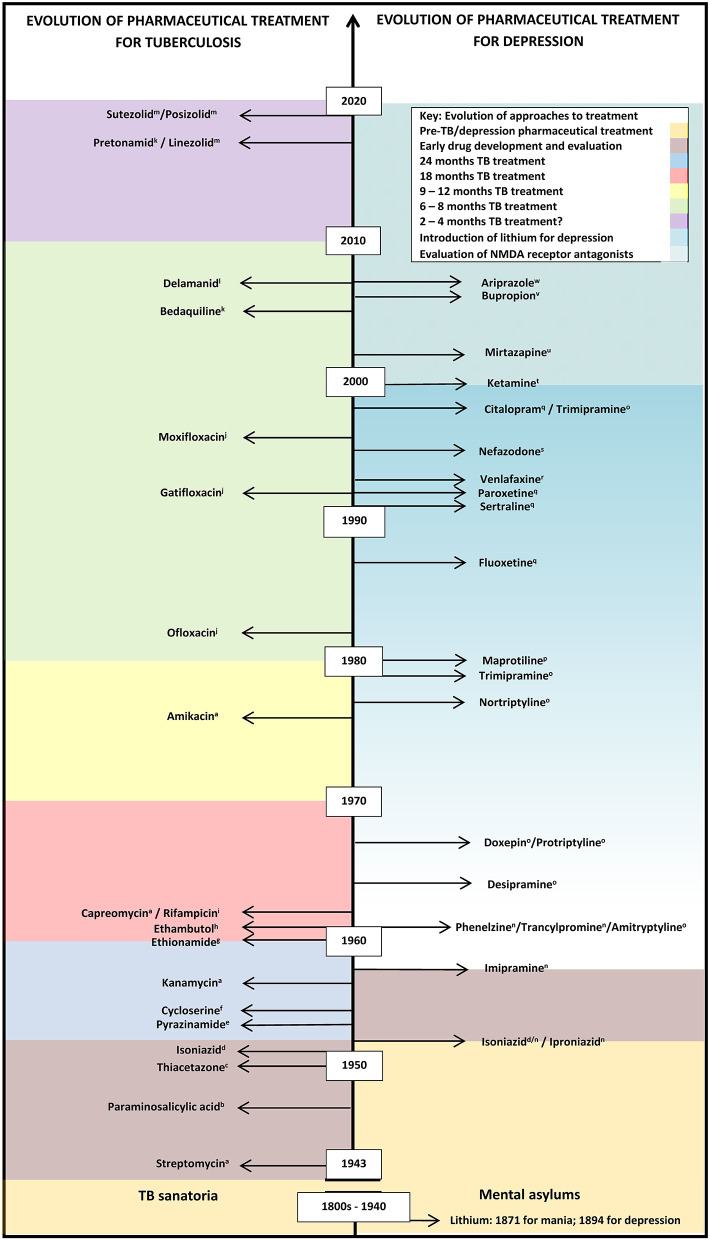
Evolution of pharmaceutical treatment for tuberculosis and depression from the pre-chemical era to recent drug trials. This information is not exhaustive. Many more classes of anti-tuberculotics have been developed compared with categories of antidepressants, with recent renewed interest in the development of antidepressants, based on the recent calculations of the growing importance of depression as a significant contribution to disease burdens. A gradual increase in lithium usage, which may be off-label in some countries, is shown by the graded coloring. *Classes of anti-tuberculotics:* ^a^Aminoglycosides; ^b^Para-aminosalicylic acid; ^c^Thiosemicarbazones; ^d^Hydrazines; ^e^Pyrazine; ^f^Serines; ^g^Thioamide; ^h^Ethylenediamines; ^i^Rifamycins; ^j^Fluoroquinolones; ^k^Diarylquinoline; ^l^Nitroimidazole; ^m^Oxazolidinone. *Categories of antidepressants:* ^n^Monoamine oxidase inhibitor (MAOI); ^O^Tricyclic antidepressants; ^p^Tetracyclic antidepressants; ^q^Selective serotonin reuptake inhibitor (SSRI); ^r^Serotonin-norepinephrine reuptake inhibitors (SNRI); ^s^Serotonin receptor antagonist with serotonin reuptake inhibition (SARI); ^t^NMDA glutamergic ionoceptor blockers; ^u^Noradrenergic α_2_-receptor antagonist with specific serotonergic receptors-2 and−3 antagonism (NASSA); ^v^Aminoketone; ^w^Atypical antipsychotic.

Para-amino-salicylic acid (PAS) for tuberculosis treatment was developed in 1940 (Figure 1), followed by streptomycin in 1943 (22, 23). PAS was moderately effective, but streptomycin showed tuberculosis is amenable to chemotherapy. Drug resistance to streptomycin emerged soon after the first patients were treated and monotherapy was stopped; PAS and streptomycin were used in combination, limiting the emergence of resistance to either drug (23). The search for new antituberculotics continued, and iproniazid/INH was introduced in 1951 (Box 1) (22). As with streptomycin, INH monotherapy resulted in resistance, guiding the introduction of combined drug regimens necessary for effective treatment (23). INH remains central to tuberculosis treatment today due to its highly bactericidal mechanism of action. Rifampicin, a rifamycin, was then developed in 1966 (Figure 1), proving highly effective combined with INH for treating drug-susceptible tuberculosis (23). Thereafter, investing in tuberculosis control in high-income countries stopped, as incidence declined and living conditions and nutrition improved (25). Tuberculosis became viewed as a disease of poverty, predominantly affecting underdeveloped countries: limited market returns detracted from new drug development.

Box 1 **The discovery and development of isoniazid (INH) (22, 24).**

Isoniazid and serendipity

Hydrazine has its origins in industrial chemistry and was produced by three chemistry pioneers, working independently. The compound was most probably originally synthesized in 1875 at the University of Berlin by Emil Fischer, who named it “hydrazine.” Pure anhydrous hydrazine was first prepared by Lobry de Bruyn in 1895, working for the German government in an effort to develop explosives from alcohol solutions. Simultaneously, between 1890 and 1894, Theodor Curtius at the University of Munich discovered hydrazine sulfate. However, further work on the new compound stopped during World War I. The antituberculotic properties were discovered by Gerhard Domagk in Germany in 1927, while testing a range compounds in animal studies to identify new antimicrobials, including, amongst others, the sulphonamides.

Yet again a war stopped further work in hydrazine. It was used by the Germans in WWII as a component of rocket fuel, and large stocks remained when the war ended, which was freely donated to civilian chemical companies interested in developing pharmaceuticals. After the war, Domagk returned to his earlier work on the sulphonamides in an attempt to find an alternative to streptomycin. Although he was unsuccessful, his work was instrumental in the eventual investigation of hydrazine in animals.

In 1950/1 two research teams working independently of each other, discovered the compound's strong antituberculotic potency. Herman Hyman Fox, working at Hoffman-La Roche in New Jersey, studied the semi-carbazones, knowing that they have antituberculotic potential. When he investigated these compounds in mice, he coincidently included some of the intermediaries, among which was INH/propiozid. Harry Yale at Squibb Institute of Medical Research in New Jersey simultaneously investigated various compounds as antituberculotic drugs in mice. Both researchers made a comparable unexpected discovery of the strong *in vivo* activity of the intermediary. After this, clinical trials were almost immediately conducted in hospitals in the New York State.

The success of INH against tuberculosis led to renewed interest for the development of other antituberculotics, eventually leading to the discovery of pyrazinamide, ethionamide and rifampicin. The history of INH highlights the speed of research: after the discovery after its antituberculotic properties, a mere 2 years of the animal tests, it was used in humans.

## Isoniazid, Serendipity, and the First Antidepressant

Monoamine oxidase was first described by Mary Hare at Oxford University in 1928, although its role in depression was not recognized until the 1950s (26). Serendipity decreed that development of antituberculous drugs would lay the foundation of psychopharmacology (22, 27). In 1950, when INH was investigated in a clinical trial of 44 patients believed to have terminal tuberculosis at Sea View Hospital, New York, unexpected positive side effects occurred (16). Patients became less depressed, engaged socially and displayed improved appetites (24). The remarkable effects of INH, coined the “miracle drug,” were documented in the news and media frenzy ensued. The Associated Press reported that the patients were “dancing in the halls tho' they had holes in their lungs,” having undergone an astonishing transformation from morbid depression to euphoria; reports completely disregarding how effective INH was for tuberculosis (16).

The mood enhancing characteristics of INH, which deaminates the biogenic amines (26), were investigated clinically among psychiatric patients by Nathan Kline in 1957, who described INH as a “psychic energizer” (27). Seventy percent of patients institutionalized for depression showed marked improvement and similar improvements were observed in ambulatory patients (27). The term “antidepressant” was coined for the mood elevating properties; by the end of the 1950s, INH was used by an estimated 400,000 psychiatric patients (27, 28).

Use of INH and iproniazid as antidepressants ended in 1961: hepatotoxicity in doses used in depressed patients halted treatment programs and INH was launched solely for treatment of tuberculosis (29). Nevertheless, this landmark event contributed to the modern view of a biological basis for psychoses, shifting the approach from management of depression as a psychodynamic process.

## Development of New Antituberculotics

The earlier successes with combination therapy for tuberculosis underwent reversal in the 1980s, when an outbreak due to *Mycobacterium tuberculosis* strains resistant to INH and rifampicin occurred in New York (30). Soon afterwards, strains with comparable resistance patterns emerged from other parts of the world (30). These patients had high mortality and limited treatment options due to the paucity of effective drugs. The simultaneous emergence of HIV in the 1980s accelerated the crisis and drug-resistant tuberculosis was recognized as a global emergency (31).

A rise in tuberculosis incidence globally continued through the 1990s and mortality rates increased, in both high-income and LMICs (2), potentially reversing all earlier gains made in tuberculosis control. A period of massive investment in research and development of new antituberculotics compounds followed (Figure 1). Academia, governments, philanthropy, industry collaborated in revising approaches to clinical testing in order to optimize time periods in which to bring these to market (32). Between 2010 and the present, investment into research and development of new antituberculotics has exceeded that of the preceding 40 years. Currently, there are a number of new compounds in development and various phases of clinical trials (33).

In contrast to the antidepressants, for the first time since 1960, two new drugs from completely new categories, bedaquiline and delaminid, became available for tuberculosis treatment in the mid-2000s (34). Both hold promise of shortening the treatment period and the potential impact on treatment may be similar to the hype that INH offered almost 70 years ago (34). A recent meta-analysis of 50 studies suggests that regimes that include bedaquiline with linezolid, later generation fluoroquinolones, clofazimine, and carbapenems were positively associated with treatment success of multidrug-resistant tuberculosis, compared with the modest results of older second-line drugs (35).

Challenges to treatment of tuberculosis have been compounded by the side effects of the many drugs' required for combination regimens to affect a cure in multidrug resistant (MDR) or extremely drug resistant (XDR) infection. These include fluoroquinolones and cycloserine/terizidone, which may cause psychiatric side effects and suicidal symptoms (36). Withdrawal of these drugs from the regimen can result in an inadequate treatment, requiring a longer duration of treatment or delaying the patients' response to treatment. Side effects of other drugs though milder, include gastro-intestinal disturbances, and peripheral neuropathy; the long duration may result in a morbid and depressed patient. Optimization of new treatment regimes, including of length of treatment, remains a priority (35).

Similar to the discovery of the anti-depressive properties of INH, it has been suggested that drugs developed for other indications may be repurposed for the management of drug resistant tuberculosis. As side effect and toxicity profiles of such compounds have been elucidated, this could hopefully hasten the introduction to the market. This includes folate inhibitors, sulfamethoxazole-trimethoprim, the antileprotic dapsone, β-lactam agents other than carbapenems and mefloquine, an antimalarial (37). Compounds developed for other purposes may also have anti-mycobacterial properties, including the anti-hypercholesterolaemia statins (*M. tuberculosis* uses cholesterol uptake in the macrophage to depress the host's immune response), antihistamines and neuroleptics including phenothiazines, anticonvulsants, anticancer drugs, such as tyrosine kinase inhibitors, and non-steroidal anti-inflammatories (37).

## Evolution of Antidepressants

INH and the related compound iproniazid (28) made two fundamental contributions to the development of psychiatry,

(1) recognition of the social health aspect to depression, changing the nature of psychiatric care of depressive patients, and
(2) the neurobiological origin of certain psychiatric conditions (38).

The MAOI activity of INH gave researchers an indispensable research tool for neurobiology and psychopharmacology (38), permitting the first aetiopathological hypothesis of any common mental disorder. The identification of the mechanisms of action of MAOIs provided the model for the next four decades for most of the present antidepressants, including a cyclopropylamine, tranylcypromine (an amphetamine analog), and phenelzine, a hydrazine derivative, which dominated the market for MAOIs by the mid-1980s (26) (Figure 1).

The second category of antidepressants developed included the tricyclic antidepressants (TCA), imipramine and related compounds (Figure 1), antihistamine derivatives preceding phenothiazines, revolutionizing the history of biological psychiatry. In a recurring theme, the first phenothiazine was synthesized in 1883 as a potential chemical dye, followed by iminodibenzyle in 1898. Proving ineffective as a dye, iminodibenzyle was shelved for 50 years, until interest in sedatives began increasing (39). Roland Kuhn, contracted by Geigy to examine the non-existent sedative effects of the iminodibenzyle derivative G 22150 in 1948, proposed its potential as an antipsychotic in 1954, but it proved too toxic for use. Geigy offered the alternative G 22355, with the homologous side chain to that on chlorpromazine in 1957. Kuhn expounded imipramine's antidepressant potential in 1958 (38, 39), generating a new era of rational drug design, in which newer antidepressants are designed to act on a particular site, receptor or enzyme (38) (Figure 1). Similar to antituberculous therapy, drugs combinations from different antidepressant categories were frequently combined, particularly for refractory cases of major depression (40). During this period the element lithium was recognized as highly efficacious in preventing depressive relapse. Initially used to control manic episodes in bipolar disorders, lithium was gradually introduced as maintenance management against repeated depressive episodes (41). The original study investigators observed that: “Instead of having a suicide rate of seven per thousand, which is the norm, we had a suicide rate of less than one per thousand”; further meta-analysis in 1999 calculated that relapse rates for major depression averaged 74% on placebo, compared with 29% on lithium (41). Focusing on manic-depression, further trials with antiepileptics, valproate and carbamazepine, between the 1960s and 1990s, which proved more successful in controlling mania in manic-depression, and lamotrigine for both manic and depressive episodes, ensued, of which only lamotrigine is FDA approved for maintenance therapy of manic depression (42).

In the late 1970s and early 1980s, the antidepressant zimelidine, was developed, an antihistamine and the first of the SSRIs. Although zimelidine was later withdrawn due to toxicity, the increase in knowledge of the mechanisms of depression led to the development of serotonin and norepinephrine (noradrenaline) reuptake inhibitors (SNRI) (Figure 1) (38). Interest in novel antidepressants thereafter waned with the recognition of “treatment intolerant” forms of depression to categories of antidepressants then available, delayed treatment responses, and unsustained remission (43).

Attention focused on chemical alteration of available drugs, reducing side-effects, promoting faster onset of action and providing more universal therapeutic action (Figure 1).

Management protocols are now being reviewed to improve understanding and address the intense social and economic burdens that accompany depression (44), although comprehensive guidelines are lacking in many countries and only 25% of guidelines identify enablers and barriers to implementation (7). Treatment of the depressed patient requires insight into depression as a medical condition, psycho-social aspects leading to poor treatment uptake, problems with treatment availability, and treatment disruption, similar to the broader view required for antituberculous treatment (43). Controlling depression will require novel chemotherapeutic approaches (45). Learning from the INH experience, re-examination and repurposing of drugs, including anesthetics, anti-epileptics, anti-hyperlipidemic statins and antibiotics may enable antidepressant research to focus on novel targets, particularly for refractory and resistant cases (46). Compounds acting on the glutamate and γ-aminobutyric acid (GABA) neurotransmitter systems, recognizing the role of these systems in major depression have been reviewed (47), based on ketamine activity (an anesthetic developed in the 1900s), which is antidepressant in low doses (48, 49). Compounds such as rapastinel and apimostinel have shown early phase II efficacy, with low toxicity (49). Other novel targets include excitatory amino acid transporter-2 (EAAT-2) reuptake enhancers and metabotropic glutamergic receptors as positive or negative modulators (47).

## The Impact of the Digital ERA

The current rate of decline of tuberculosis incidence remains too slow and it could potentially remain a major contributor to infectious disease burdens beyond 2050 (50), until the 22nd century (51). The World Health organization has set the “End Tuberculosis Strategy” goal to eliminate tuberculosis by 2035, requiring vast investments in the discovery and development of new tools for tuberculosis control, including an effective vaccine (52), but wider understanding of the patient as an individual, obstacles to successful management and why treatment failures occur, and addressing the economic and social determinants of disease, are of equal importance (51, 53). The Global Plan to Stop Tuberculosis estimated that the funding gap to realize the 2035 elimination target at USD1.3 billion (51).Development of new drugs for tuberculosis and new drug design has benefited from computational tools for rational drug design and identification of new drug targets, and digital health solutions hold promise for new antituberculotics (32, 37, 54). Mobile phone communications between treatment provider and tuberculosis patients can be used to customize patient support, without the patient having to come to the clinic (55). Electronic pill box dispensers already monitor if patients access treatment as prescribed, and if not, prompt care providers to intervene timeously before treatment is abandoned (55). Deep learning has permitted the identification of novel compounds. A pioneering deep learning algorithm has identified a new broad-spectrum antimicrobial, halicin, that showed killing of *M. tuberculosis* at 16 μg/mL (56), named for the computer HAL in “2001: A Space Odyssey” (57). The novel mechanism of action suggests currently recognized resistance mechanisms would not affect halicin's antimicrobial potential (56).

The current outlook for depression remains concerning, as global disease burdens increase, compounded by the paucity of management guidelines (7): treatment demands will likely grow, necessitating innovative solutions. Potentiation of currently available bioactive compounds through methylation, altering the solubility, could enhance binding affinity and drug metabolism up to a factor of 2,000, permitting use of lower drug dosages: reduced side effects and improved safety profiles should decrease default rates (58). Inexpensive novel techniques have been successfully used for citalopram, an antidepressant, and tedizolid (58), an oxazolidinone with significant antituberculotic activity (59). Improved computational power and artificial intelligence to mine health information data sets for depression may reveal genetic predisposition, psychiatric sub-types and identify new biochemical pathways for treatment previously unrecognized with classical approaches (60). Mobile-health interventions enable confidential customized treatment and patient support (61–63). Self-help applications may prevent depression, prior to its development into full disease, decreasing demands on health resources (60, 62, 63). Deep learning algorithms targeting single nucleotide polymorphisms as genetic biomarkers may predict clinical treatment outcomes and adverse drug reactions in patients with major depressive disorder treated with antidepressants (64).

## Discussion

The right of everyone to greater standards of mental and physical health has been emphasized at a high-level United Nations meeting on tuberculosis in 2018 (51). Patient-centered care of tuberculosis and depression requires multidisciplinary approaches for integrated management of risk factors and co-morbidities (7, 53, 64). A recent study showed only five of 26 countries, 21 of which are high tuberculosis-incidence, had standard protocols for the co-management of other disorders: supposed barriers to program integration included limited capacity, not perceiving mental health as problematic, resource insufficiencies and social stigmata of tuberculosis and mental disease (65), despite mounting evidence of the association internationally (8, 66–71), possibly accounting for the lack of comprehensive guidelines for addressing comorbidities with depression identified in LMICs (7). Nonetheless, integration of HIV and tuberculosis programs has greatly reduced patient morbidity and mortality (53); integration of diabetes with tuberculosis programs improved treatment outcomes of both (72).

## Conclusion

As treatments evolve, big data and mobile health technologies should facilitate easier monitoring of potential drug interactions–beneficial or detrimental–to optimize therapies. Integrating tuberculosis management and mental health within health systems can and should be designed according to patient needs (8, 65). In the absence the urgent introduction of policies permitting patient-centered management of the syndemic, the public health implications are grave and the global outlook for both diseases remains bleak.

## Author Contributions

MV conceptualized paper. Both authors contributed to the writing of the paper and approved the final submission.

## Conflict of Interest

MV and KK are employed by the South African Medical Research Council.
